# Mortality with musculoskeletal disorders as underlying cause in Sweden 1997-2013: a time trend aggregate level study

**DOI:** 10.1186/s12891-016-1024-9

**Published:** 2016-04-14

**Authors:** Aliasghar A. Kiadaliri, Martin Englund

**Affiliations:** Lund University, Faculty of Medicine, Department of Clinical Sciences Lund, Orthopaedics, Clinical Epidemiology Unit, Lund, Sweden; Health Services Management Research Center, Institute for Futures Studies in Health, Kerman University of Medical Sciences, Kerman, Iran; Clinical Epidemiology Research and Training Unit, Boston University School of Medicine, Boston, MA USA; Clinical Epidemiology Unit, Skåne University Hospital, Klinikgatan 22, SE-221 85 Lund, Sweden

**Keywords:** Mortality, Musculoskeletal disorders, Temporal trend, Sweden

## Abstract

**Background:**

The aim was to assess time trend of mortality with musculoskeletal disorders (MSD) as underlying cause of death in Sweden from 1997 to 2013.

**Methods:**

We obtained data on MSD as underlying cause of death across age and sex groups from the National Board of Health and Welfare's Cause of Death Register. Age-standardized mortality rates per million population for all MSD, its six major subgroups, and all other ICD-10 (International Classification of Disease) chapters were calculated. We computed the average annual percent change (AAPC) in the mortality rates across age/sex groups using joinpoint regression analysis by fitting a regression line to the natural logarithm of the age-standardized mortality rates and calendar year as a predictor.

**Results:**

There were a total of 7 976 deaths (0.5 % of all causes deaths) with MSD as the underlying cause of death (32.5 % of these deaths caused by rheumatoid arthritis [RA]). The overall age-standardized mortality rates (95 % CI) were 16.0 (15.4 to 16.7) and 24.9 (24.1 to 25.7) per million among men and women, respectively (women/men rate ratio 1.55; 95%CI 1.47 to 1.63). On average, mortality rate declined by 2.3 % per year and only circulatory system mortality had a more favourable decline than mortality with MSD as underlying cause. Among MSD the highest decline was observed in RA (3.7 % per year) during study period. Across age groups, while there were generally stable or declining trends, spondylopathies and osteoporosis mortality among people ≥ 75 years increased by 2 and 1.5 % per year, respectively.

**Conclusion:**

In overall, mortality with MSD as underlying cause has declined in Sweden over last two decades, with the highest decline for RA. However, there are variations across MSD subgroups which warrants further investigations.

## Background

Musculoskeletal disorders (MSD) cover a wide range of disorders affecting joints, bones, muscles and soft tissues and are considered as the most common cause of severe long term pain and physical disability [1]. MSD are highly prevalent worldwide, and in a steadily aging population with increased prevalence of obesity and reduced physical activity, the prevalence of many of MSD will increase in coming years [2]. Globally, 21.3 % of years lived with disability was attributed to MSD in 2010 (44.7 % increase from 1990) [3]. MSD were considered as the fourth (third in developed countries) greatest contributor in disability-adjusted life years (DALYs) in 2010 [4].

In spite of substantial burden of MSD on individuals and societies, there is a lack of adequate recognition at the level of policy-making or priority [5]. In response to this, the Bone and Joint Decade 2000–2010 was endorsed by the United Nations and the World Health Organization [6]. While less attention had been paid to mortality associated with MSD, a growing recognition of MSD-related mortality has been emerged in recent years [7–9]. Recent evidence suggests that MSD including rheumatoid arthritis (RA) and osteoarthritis (OA) are associated with excess all-cause and disease-specific mortality [10–15]. In the Global Burden of Disease Study, MSD were considered as underlying cause of 153 500 deaths worldwide in 2010 (about 0.3 % of all causes deaths) and age-standardized mortality rate increased from 17 per million in 1990 to 23 per million in 2010 (a 37.8 % increase) [16]. It should be noted that these numbers are underestimated because MDS are likely underreported on death certificates especially as underlying cause of death [5, 17].

Although, the accuracy of death certificates has been questioned [17–19], these are the main source of mortality data available for whole populations at often national level, and they are widely used in cause of death analyses. While a few studies have investigated time trend of a specific MSD such as RA [8, 20, 21] and systemic lupus erythematosus [22–24], updated information on mortality is needed, for instance due to the introduction of new biological drugs in the treatments of many inflammatory rheumatic diseases. Also, to our best knowledge, no previous study has investigated all mortality associated with MSD and its major subgroups in a single study using the same data source and uniform methodology. Thus, our aim was to provide an up-to-date data on the recent trend of mortality with MSD and its six subgroups as underlying cause in Sweden during 1997–2013. Such data not only provide tools to monitor progress towards national public health goals but also helps to evaluate interventions, to identify high risk population subgroups, and to make future projections.

## Methods

### Data sources

Data on population across sex and age groups were collected from the Statistics Sweden (http://www.scb.se). Data on mortality were obtained from the National Board of Health and Welfare's Cause of Death Register which includes all those who died during one calendar year and were registered in Sweden at the time of death, regardless of whether the death occurred inside or outside the country. The causes of death are coded centrally at the Statistics Sweden according to the International Classification of Diseases, the 10^th^ revision (ICD-10). The Cause of Death Register contains a single underlying cause of death, up to 48 additional contributory causes of death, and demographic data. For this study we used publicly available data which includes only the underlying cause of death by sex, age, region, and year (http://www.socialstyrelsen.se/statistics/statisticaldatabase/causeofdeath). MSD were identified as ICD-10 codes M00-M99. In addition, six major subcategories of MSD with higher mortality rates were identified: pyogenic arthritis (M00), RA (M05-M06), OA (M15-M19), systemic connective tissue disorders (M30-M35), spondylopathies (M45-M48), and osteoporosis (M80-M81). To compare the trend in mortality with MSD as underlying cause with trend in mortality from other diseases over the study period, we also included fifteen other ICD-10 chapters (Table 3 in Appendix). Due to very low mortality over study period, the following ICD-10 chapters were excluded: Diseases of the eye and adnexa (H00-H59); Diseases of the ear and mastoid process (H60-H95); and Pregnancy, childbirth and the puerperium (O00-O99).

### Statistical analysis

Age-standardized mortality rates per 1 million population were calculated by means of direct standardization using the WHO Reference Population [25]. These age-standardized rates were calculated across age and sex groups. We also computed women to men age-standardized rate ratio and its 95 % confidence interval [26]. The percent change was calculated as the difference between the average age-standardized rate of the last two years and the average rate of the first two years divided by the average rate the first two years. Time trends in age-standardized mortality rate were analyzed using joinpoint regression. This was done using the Joinpoint Regression Program version 4.2.0.2 from the Surveillance Research Program of the US National Cancer Institute (http://surveillance.cancer.gov/joinpoint). Joinpoint regression identifies points with a significant change in trend (“joinpoints”) and determined linear trends between joinpoints. In the software a series of permutation tests proposed by Kim et al. [27] is applied to compute the number of joinpoints to best fit the data. For each joinpoint an annual percentage change (APC) is estimated by fitting a regression line to the natural logarithm of the age-standardized rates, using calendar year as a predictor. The average annual percent change (AAPC) as the weighted average of APCs was computed to provide a summary measure of the trend for the whole time period [28]. We used the empirical quantile method with 1 000 resamples to calculate 95 % confidence interval of AAPC. Since the empirical quantile method is not available for comparison between groups, we used parametric method to compare AAPC of MSD mortality with other ICD-10 chapters. In trend analysis across age groups, due to low number of death in MSD subcategories, we smoothed the mortality rates using a four-year moving average.

It has been suggested that several MSD should not be considered as underlying cause of death (defined as “garbage codes”, Table 4 in Appendix) [29]. In a sensitivity analysis, we calculated the AAPC excluding deaths attributed to these causes. It should be noted that across the MSD subcategories, excluding these “garbage codes” only influenced OA and spondylopathies. In addition, because all OA codes (M15-M19) are considered as “garbage codes”, we did our sensitivity analysis only on all MSD and spondylopathies.

## Results

**Fig. 1:**
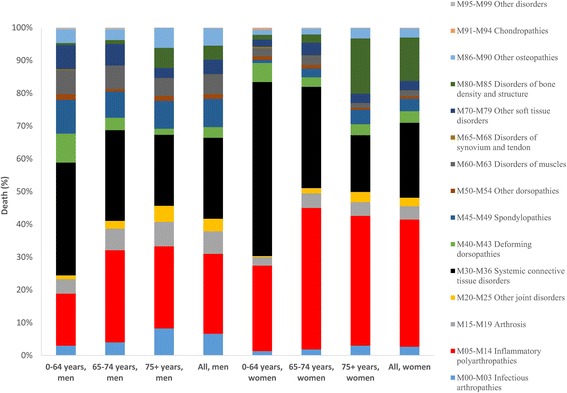
Percentage of death due to musculoskeletal disorders by sex, age and cause in Sweden, 1997–2013

During 1997 to 2013, 2 411 men and 5 565 women had a MSD registered as their underlying cause of death in Sweden (0.3 and 0.7 % of all causes mortality among men and women, Table 5 in Appendix). In overall, among MSD, RA was the leading cause of death (32.5 % all deaths) followed by systemic connective tissue disorders (23.4 % of all deaths). Across age and sex groups, while RA was the leading cause of death for men and women aged 65 years and older, among younger people systemic connective tissue disorders were associated with the highest number of deaths (45.9 % of all death in this age group, Fig. 1).

The overall age-standardized mortality rates (95 % CI) were 16.0 (15.4 to 16.7) and 24.9 (24.1 to 25.7) per million among men and women, respectively. This figures corresponded to a statistically significant women/men rate ratio (95 % CI) of 1.55 (1.47 to 1.63). Across MSD subcategories, while women had statistically significantly a higher age-standardized mortality rate for RA, systemic connective tissue disorders, and osteoporosis as underlying cause compared to men, opposite was observed for pyogenic arthritis and spondylopathies (Table 1).

**Table 1:** Overall age-standardized mortality rate of musculoskeletal disorders as underlying cause per million people in Sweden, 1997–2013

ICD title (codes)	Women	Men	Women/men rate ratio
All musculoskeletal disorders (M00-M99)	24.9 (24.1 to 25.7)	16.0 (15.4 to 16.7)	1.6 (1.5 to 1.6)
Pyogenic arthritis (M00)	0.56 (0.46 to 0.67)	0.94 (0.79 to 1.09)	0.60 (0.47 to 0.76)
Rheumatoid arthritis (M05-M06)	8.9 (8.5 to 9.4)	3.4 (3.2 to 3.7)	2.6 (2.4 to 2.9)
Osteoarthritis (M15-M19)	0.94 (0.80 to 1.1)	1.0 (0.88 to 1.2)	0.90 (0.73 to 1.1)
Systemic connective tissue disorders (M30-M35)	7.3 (6.8 to 7.7)	4.2 (3.9 to 4.6)	1.7 (1.6 to 1.9)
Spondylopathies (M45-M48)	0.75 (0.64 to 0.87)	1.4 (1.2 to 1.6)	0.55 (0.45 to 0.67)
Osteoporosis (M80-M81)	2.1 (1.9 to 2.2)	0.45 (0.35 to 0.54)	4.7 (3.7 to 5.9)

**Fig. 2:**
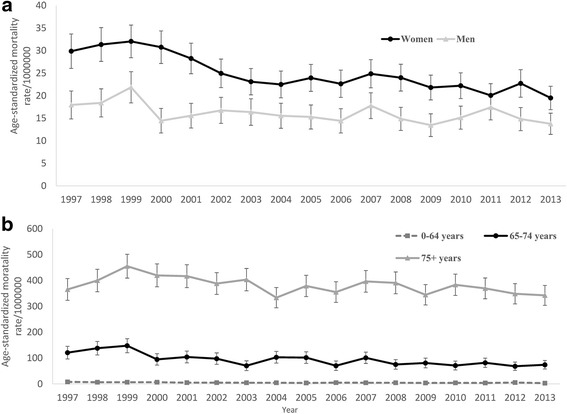
Age-standardized mortality rate (95 % CI) of musculoskeletal disorders as underlying cause across sex and age groups in Sweden, 1997–2013

The age-standardized mortality rate of MSD as underlying cause for Swedish population declined from 24.7 per million in 1997 to 17.2 per million in 2013 (a percent change of −27.8 %, Fig. 2a). While women had higher mortality rates than men in all study years, the disparity was declining over time. Across age groups, while age-standardized rate substantially declined in 2013 compared to 1997 among younger age groups (the percent change of −42.2 and −45.0 % in 0–64 and 65–74 years groups, respectively), a smaller decline of 9.7 % was observed among oldest age group (from 365.3 per million in 1997 to 342.3 per million in 2013, Fig. 2b).

**Fig. 3:**
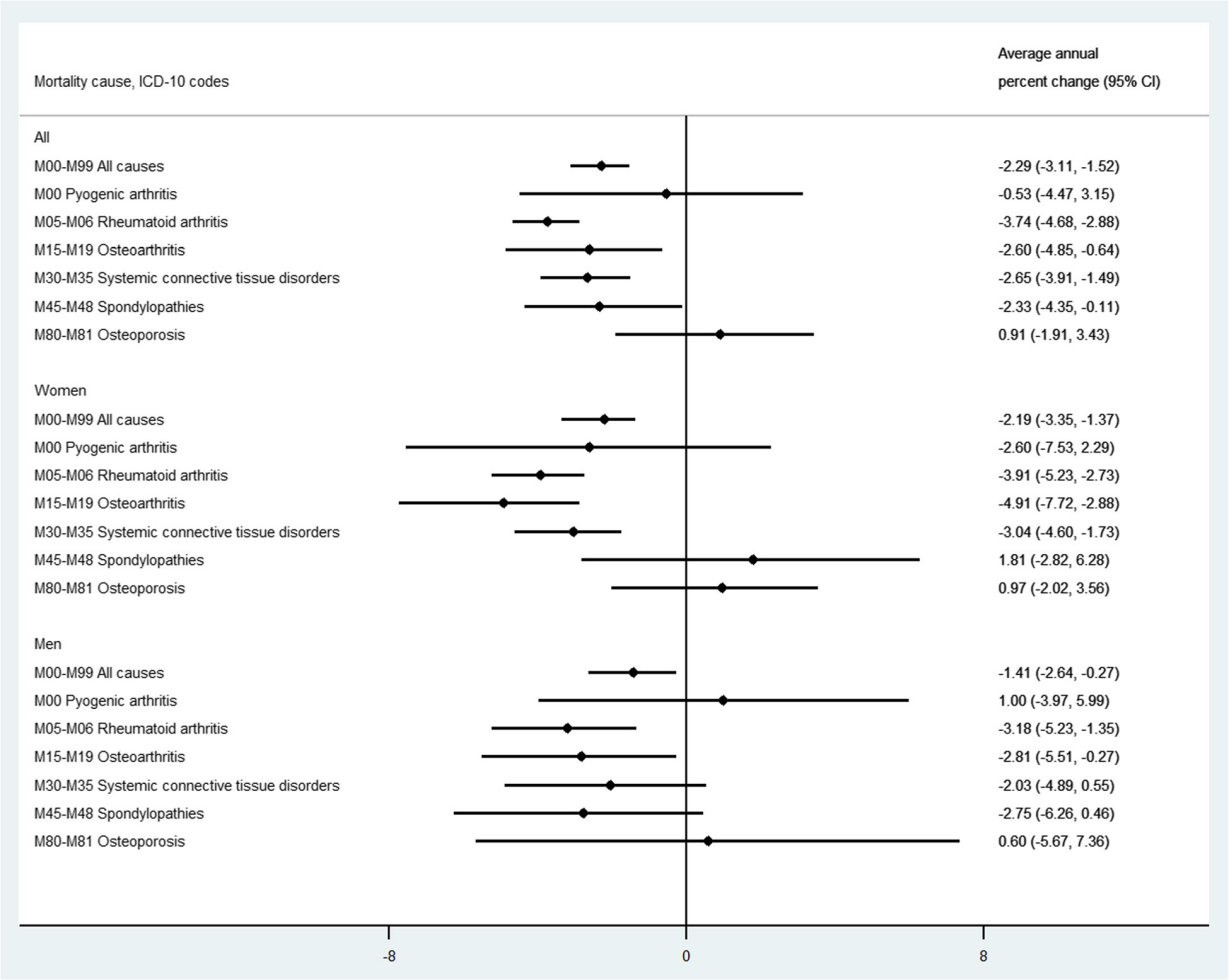
Average annual percent change of mortality with musculoskeletal disorders as underlying cause in Sweden obtained from joinpoint regression analysis 1997–2013

On average, the age-standardized mortality rate declined by 2.3 % (95 % CI: 1.5 to 3.1) annually during the study period in Sweden (Fig. 3). Among MSD subcategories while there were statistically significant declining trend for RA, OA, systemic connective tissue disorders, and spondylopathies, the trends for pyogenic arthritis and osteoporosis were stable during 1997–2013. Among ICD-10 chapters, only diseases of the circulatory system (I00-I99) had a more profound reduction than MSD (Table 2). There was no statistically significant difference in AAPC of MSD and four other chapters (i.e., diseases of the respiratory system (J00-J99), diseases of the genitourinary system (N00-N99), certain conditions originating in the perinatal period (P00-P96), and congenital malformations, deformations and chromosomal abnormalities (Q00-Q99)).

**Table 2:** Annual percent change and average annual percent change in age-standardized mortality rates for ICD-10 chapters, 1997–2013

ICD-10 chapter (codes)	Age-standardized mortality rate per million people		Period	APC, %	AAPC, %	Mean difference (95 % CI) in AAPC compared with MSD (M00-M99)
	1997	2013				
Certain infectious and parasitic diseases (A00-B99)	45.2	85.3	1997–2013	4.25***	4.25***	6.54 (5.71 to 7.37)
Neoplasms (C00-D48)	1270.7	1084.9	1997–2005	−0.57***	−0.92***	1.34 (0.72 to 2.03)
			2005–2010	−1.73***		
			2010–2013	−0.49		
Diseases of the blood and blood-forming organs and certain disorders involving the immune mechanism (D50-D89)	10.0	11.3	1997–2013	0.61	0.61	2.90 (1.88 to 3.91)
Endocrine, nutritional and metabolic diseases (E00-E90)	104.4	102.6	1997–2005	0.97	−0.15	2.15 (1.22 to 3.07)
			2005–2013	−1.24*		
Mental and behavioural disorders (F00-F99)	141.7	184.4	1997–1999	11.54	1.67	3.97 (1.87 to 6.06)
			1999–2010	−0.61		
			2010–2013	3.89		
Diseases of the nervous system (G00-G99)	99.9	159.6	1997–2013	3.39***	3.39***	5.69 (4.93 to 6.44)
Diseases of the circulatory system (I00-I99)	2038.4	1202.0	1997–2002	−2.83***	−3.27***	−0.98 (−1.76 to −0.20)
			2002–2005	−4.55**		
			2005–2013	−3.07***		
Diseases of the respiratory system (J00-J99)	303.4	227.9	1997–2010	−2.71***	−1.65**	0.64 (−0.69 to 1.98)
			2010–2013	3.06		
Diseases of the digestive system (K00-K93)	141.7	120.3	1997–2006	−0.01	−1.30**	0.99 (0.01 to 1.98)
			2006–2013	−2.92**		
Diseases of the skin and subcutaneous tissue (L00-L99)	7.0	6.3	1997–2013	−0.53	−0.53	1.76 (0.58 to 2.94)
Diseases of the musculoskeletal system and connective tissue (M00-M99)	24.7	17.2	1997–2013	−2.29***	−2.29***	-
Diseases of the genitourinary system (N00-N99)	54.2	38.5	1997–2013	−2.80***	−2.80***	−0.51 (−1.37 to 0.36)
Certain conditions originating in the perinatal period (P00-P96)	23.1	21.9	1997–2003	5.40*	−0.58	1.71 (−3.28 to 6.70)
			2003–2006	−14.93		
			2006–2013	1.09		
Congenital malformations, deformations and chromosomal abnormalities (Q00-Q99)	36.5	25.4	1997–2013	−3.14***	−3.14***	−0.85 (−1.82 to 0.13)
Symptoms, signs and abnormal clinical and laboratory findings, not elsewhere classified (R00-R99)	95.7	123.2	1997–2007	1.09	0.85	3.14 (0.05 to 6.23)
			2007–2010	13.79		
			2010–2013	−11.30**		
External causes of morbidity and mortality (V01-Y98)	338.6	309.3	1997-2013	−0.78*	−0.78*	1.51 (0.71 to 2.31)

*** *P*< 0.001; ** *P*< 0.01; * *P*< 0.05

*APC* annual percent change, *AAPC* average annual percent change, *MSD* musculoskeletal disorders

**Fig. 4:**
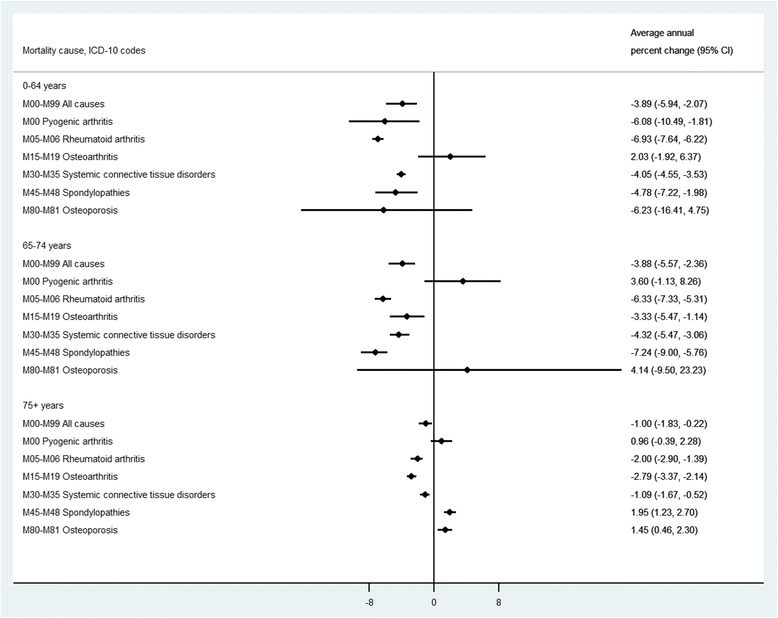
Average annual percent change of mortality with musculoskeletal disorders as underlying cause in Sweden obtained from joinpoint regression analysis, by age groups 1997–2013. Footnote: The mortality rates for subgroups of musculoskeletal disorders are four-year moving average rates

Our subgroup analyses across age and sex groups revealed that among men and women the highest decline was observed in RA (3.2 % per year) and OA (4.9 % per year), respectively. While women experienced a statistically significant decline in mortality with systemic connective tissue disorders as underlying cause, this was relatively constant among men (AAPC = −2.0 %, *P* = 0.20). Across age groups, the oldest age group (75+ years) experienced less profound changes in mortality with MSD as underlying cause (Fig. 4). While people aged 0–64 experienced a statistically significant decline in mortality with pyogenic arthritis as underlying cause, the trend was constant among older people. On the other hand, the trend in mortality with OA as underlying cause was constant among people aged 0–64 years and declining among older age groups. In addition, while mortality with spondylopathies and osteoporosis as underlying cause were either declining or constant among younger age groups (i.e., 0–64, and 65–74 years), these have increased among the oldest age group during the study period. Of course, mortality rate of osteoporosis as underlying cause steadily declined by 1.2 % per year since 2001 but a sharp increase of 10.9 % per year during 1997–2001 resulted in an overall increasing trend for this subgroup.

A total of 1 543 (19.3 %) of deaths with MSD as underlying cause were attributed to garbage codes (25.7 and 16.6 % of deaths among men and women, respectively, Table 5 in Appendix). Excluding these potential garbage codes had negligible impact on AAPC of mortality with MSD as underlying cause among women and in total population (among women AAPC changed from −2.2 to −2.7 % and in total population from −2.3 to −2.1 %). However, excluding these garbage codes resulted in a statistically non-significant AAPC of −0.9 % (*P* = 0.21) among men. In addition, across age groups, the declining trend for mortality with MSD as underlying cause among people aged ≥ 75 years was no longer statistically significant (AAPC = −0.5 %, *P* = 0.45). For mortality with spondylopathies as underlying cause, the exclusion of garbage codes only influenced the trend for the total population and it became no longer statistically significant (AAPC = −0.3 %, *P* = 0.88).

## Discussion

In the current study, we have presented an up-to-date data on recent trends in mortality with MSD and its six major subcategories as underlying cause in Sweden. Our results showed that MSD were recorded as underlying cause of death on 0.5 % of all death certificates during 1997–2013. We found evidence that mortality with MSD as underlying cause declined during the study period and its annual decline was generally more profound than for other disease categories. Although the age-standardized mortality rates of MSD as underlying cause declined in both men and women, our subgroup analyses revealed important variations in MSD subcategories across age and sex groups. In addition, while women had higher mortality rates compared with men, this gender disparity was declining over time.

Comparing the recent (2007–2013) mortality with MSD as underlying cause in Sweden with Denmark (http://www.statbank.dk/), Australia (http://www.aihw.gov.au/deaths/grim-books/) and USA (http://wonder.cdc.gov/ucd-icd10.html) showed that while the proportion of MSD from all-cause mortality was similar across countries (0.5 % in Sweden, 0.6 % in USA, and 0.8 % in Denmark and Australia), Sweden had the lowest age-standardized mortality rate (pooled age-standardized mortality rate per million people: Sweden 21.1, USA 26.3, Denmark 32.9, and Australia 26.4). The differences in sociodemographic status, health care system, and mortality coding practices might be potential reasons for observed difference in mortality rates across these countries.

We found that mortality with MSD its four subcategories (i.e. RA, OA, systemic connective tissue disorders, and spondylopathies) as underlying cause statistically significantly declined in Sweden over the study period. This finding is in line with declining trend in mortality from RA, systemic lupus erythematosus, ankylosing spondylitis and polyarteritis nodosa in France [8], Spain [24], and England [30]. Better management of MSD induced by advancement in pharmacological interventions and medical procedures might explain this observed declining trend. For example, a recent study showed that time to initiation disease modifying antirheumatic drug has substantially shortened over the past four decades among people with RA [31]. Nevertheless, analysis across age groups revealed an increase in mortality with spondylopathies and osteoporosis as underlying cause among the oldest (aged ≥ 75 years). This might be due to aging population which has increased number of older people. Moreover, promoted awareness of the severity of these conditions in recent years might have increased reporting them as a cause of death [32]. Further research is required to explain these increasing trends among the most elderly.

The mortality rates of MSD and its three subcategories (i.e., RA, systemic connective tissue disorders, and osteoporosis) as underlying cause were higher among women compared to men and this is mainly attributed to higher prevalence of these disorders among women [33, 34]. Previous studies have suggested similar gender inequality for RA [8, 20, 21], dermatomyositis and polymyositis [35], and systemic lupus erythematosus [22, 24]. On the other hand, men had higher mortality rates of pyogenic arthritis and spondylopathies as underlying cause compared with women. The similar pattern for ankylosing spondylitis was observed in England [30]. This might be due to higher prevalence of these disorders among men and also difference in severity of disease. For example, previous studies reported a higher prevalence of ankylosing spondylitis, lumbar spondylosis, and cervical spondylosis among men [36–38] and a tendency to have more severe ankylosing spondylitis compared with women [39]. While, these gender gaps were generally closing over time, more efforts are required for further reduction. For example, if we naively assume that the projected trends in mortality with MSD as underlying cause will be observed during next 10 years, then AAPC for women should be doubled during this period (i.e., increase from −2.2 to −4.7 %) in order to close observed gender gap in our study.

The limitations of the current study should also be considered when interpreting its findings. We only analyzed mortality due to underlying cause of death which suffers from underestimation for MSD as these are not usually considered as underlying cause of death [5]. Inaccuracy and errors in completion of death certificate are another potential limitation of our study, but is unlikely to have substantially changed over time [18]. The small number of deaths, especially for MSD subgroups, might have limited the power of our study to detect significant joinpoints during the study period. In addition, because our study was an ecological study with no individual-level data available, we were unable to adjust for any potential confounders in addition to age and sex. Also, we cannot further examine factors influencing the observed trend in mortality with MSD as underlying cause. In spite of these limitations, the current study have important implications including new insight about recent temporal trends in mortality with MSD as underlying cause of death which might be used to predict mortality rates in coming years in Sweden. The findings can be used to develop a hypothesis that increased use of biological treatments in e.g. RA has impacted on mortality associated with MSD. In addition, the observed increasing trends in mortality with spondylopathies and osteoporosis as underlying cause require further attention.

## Conclusion

The mean age-standardized mortality rate with MSD as underlying cause was 21.1 per million people per year in Sweden during 1997–2013. The present study indicated that mortality with MSD as underlying cause declined by 2.3 % per year in Sweden and this reduction was generally more favourable than other ICD-10 chapters. However, we found variations in time trends for MSD subcategories across sex and age groups. Our findings revealed that older people (aged ≥ 75 years) observed an increasing trend in mortality with spondylopathies and osteoporosis as underlying cause which warrants further investigations. Moreover, while mortality rates of MSD and its three subcategories (RA, systemic connective tissue disorders, and osteoporosis) as underlying cause were higher among women, more Swedish men died from pyogenic arthritis and spondylopathies during the study period. Analyzing time trend in MSD mortality using multiple cause of death framework and using individual-level data are subjects for future studies.

### Availability of supporting data

All relevant raw data are freely available from the Swedish Cause of Death Register (http://www.socialstyrelsen.se/statistics/statisticaldatabase/causeofdeath).

### Ethics approval and consent to participate

Not applicable (publicly available data were used).

## Appendix

**Table 3:** The ICD-10 chapters included in the study for comparison

ICD-10 title	ICD codes
Certain infectious and parasitic diseases	A00-B99
Neoplasms	C00-D48
Diseases of the blood and blood-forming organs and certain disorders involving the immune mechanism	D50-D89
Endocrine, nutritional and metabolic diseases	E00-E90
Mental and behavioural disorders	F00-F99
Diseases of the nervous system	G00-G99
Diseases of the circulatory system	I00-I99
Diseases of the respiratory system	J00-J99
Diseases of the digestive system	K00-K93
Diseases of the skin and subcutaneous tissue	L00-L99
Diseases of the genitourinary system	N00-N99
Certain conditions originating in the perinatal period	P00-P96
Congenital malformations, deformations and chromosomal abnormalities	Q00-Q99
Symptoms, signs and abnormal clinical and laboratory findings, not elsewhere classified	R00-R99
External causes of morbidity and mortality	V01-Y98

**Table 4:** Musculoskeletal disorders considered as garbage codes and excluded in a sensitivity analysis

ICD-10 title	ICD codes	Total number of deaths 1997-2013	
		Men	Women
Postinfective and reactive arthropathies in diseases classified elsewhere	M03	0	0
Psoriatic and enteropathic arthropathies	M07	0	0
Juvenile arthritis in diseases classified elsewhere	M09	0	0
Gout	M10	19	28
Other crystal arthropathies	M11	2	3
Other specific arthropathies	M12	0	0
Arthropathies in other diseases classified elsewhere	M14	0	0
Osteoarthritis	M15-M19	165	227
Other joint disorders	M20-M25	93	145
Kyphosis and lordosis	M40	14	52
Spondylosis	M47	9	5
Other spondylopathies	M48	81	154
Spondylopathies in diseases classified elsewhere	M49	0	0
Cervical disc disorders	M50	5	6
Other intervertebral disc disorders	M51	10	6
Other dorsopathies, not elsewhere classified	M53	4	5
Dorsalgia	M54	15	23
Myositis	M60	45	27
Disorders of muscle in diseases classified elsewhere	M63	0	0
Disorders of synovium and tendon	M65-M68	1	4
Soft tissue disorders related to use, overuse and pressure	M70	11	7
Other bursopathies	M71	9	2
Soft tissue disorders in diseases classified elsewhere	M73	0	0
Shoulder lesions	M75	0	0
Enthesopathies of lower limb, excluding foot	M76	0	0
Other enthesopathies	M77	0	0
Other soft tissue disorders, not elsewhere classified	M79	27	105
Osteomyelitis	M86	104	112
Other disorders of the musculoskeletal system and connective tissue	M95-M99	5	13

**Table 5:** Number of death across musculoskeletal disorders subgroups by sex and year

ICD-10 title (codes)		1997	1998	1999	2000	2001	2002	2003	2004	2005	2006	2007	2008	2009	2010	2011	2012	2013
Infectious arthropathies (M00-M03)	Men	13	6	9	4	6	10	11	4	13	5	10	5	9	15	11	13	15
	Women	9	6	15	15	7	6	5	8	8	9	3	12	7	11	13	6	7
Inflammatory polyarthropathies (M05-M14)	Men	41	35	37	27	41	37	44	37	33	34	41	26	26	24	37	32	36
	Women	127	137	159	151	161	118	133	108	128	131	126	125	134	107	110	113	90
Osteoarthritis (M15-M19)	Men	6	12	13	13	8	11	10	11	9	12	8	6	10	10	9	8	9
	Women	18	16	16	13	13	13	8	12	13	14	13	14	15	8	16	13	12
Other joint disorders (M20-M25)	Men	14	18	13	9	3	2	0	4	2	3	0	2	3	6	4	5	5
	Women	15	29	30	11	8	5	4	4	6	2	5	2	1	6	4	5	8
Systemic connective tissue disorders (M30-M36)	Men	34	40	43	31	29	42	38	28	33	28	37	43	30	41	45	24	30
	Women	79	81	91	90	82	73	73	79	74	65	72	79	71	65	63	70	65
M40-M43 Deforming dorsopathies (M40-M43)	Men	2	2	3	4	2	4	4	8	5	8	10	1	6	5	2	7	5
	Women	5	11	9	10	11	17	13	10	6	13	14	16	13	17	13	9	10
Spondylopathies (M45-M49)	Men	18	15	11	10	13	7	10	18	11	8	12	15	11	8	10	20	11
	Women	9	11	18	11	6	15	9	7	11	8	11	11	12	17	21	13	21
Other dorsopathies (M50-M54)	Men	1	1	3	1	5	0	3	3	3	0	3	3	3	3	0	1	1
	Women	3	1	9	0	3	2	1	3	5	0	3	2	2	1	2	2	1
Disorders of muscles (M60-M63)	Men	0	4	10	4	3	8	6	4	8	11	15	14	11	16	17	11	5
	Women	2	1	4	9	3	4	6	2	6	7	9	3	6	8	11	7	9
Disorders of synovium and tendon (M65-M68)	Men	0	0	0	0	1	0	0	0	0	0	0	0	0	0	0	0	0
	Women	1	0	0	0	0	0	1	1	1	0	0	0	0	0	0	0	0
Other soft tissue disorders (M70-M79)	Men	2	7	12	2	10	8	3	3	5	4	9	9	4	2	7	9	10
	Women	16	16	4	12	6	8	11	10	9	4	18	11	8	8	7	8	7
Disorders of bone density and structure (M80-M85)	Men	1	2	5	7	6	6	4	3	11	5	11	11	6	7	8	5	6
	Women	29	31	34	37	43	54	45	32	48	45	53	50	38	58	30	53	57
Other osteopathies (M86-M90)	Men	7	5	11	5	4	5	3	7	4	8	8	6	3	12	20	9	9
	Women	10	16	3	10	11	5	4	13	10	6	9	7	3	12	12	11	9
Chondropathies (M91-M94)	Men	0	0	0	0	0	0	0	0	0	0	0	0	0	0	0	1	0
	Women	0	1	0	0	0	0	0	0	0	1	0	0	0	1	0	0	0
Other disorders of the musculoskeletal system and connective tissue (M95-M99)	Men	0	0	1	1	0	1	1	0	0	0	0	0	1	0	0	0	0
	Women	0	1	1	2	0	3	0	0	0	0	0	1	3	1	0	0	1
Musculoskeletal disorders (M00-M99)	Men	139	147	171	118	131	141	137	130	137	126	164	141	123	149	170	145	142
	Women	323	358	393	371	354	323	313	289	325	305	336	333	313	320	302	310	297

## Abbreviations

AAPC: average annual percent change
APC: annual percent change
CI: confidence interval
DALYs: disability-adjusted life years
ICD-10: International Classification of Disease, the10th revision
MSD: musculoskeletal disorders
OA: osteoarthritis
RA: rheumatoid arthritis
WHO: World Health Organization
